# RNA-seq Insights Into the Impact of *Alteromonas macleodii* on *Isochrysis galbana*

**DOI:** 10.3389/fmicb.2021.711998

**Published:** 2021-09-08

**Authors:** Jia-Yi Cao, Ying-Ying Wang, Min-Nan Wu, Zhou-Yan Kong, Jing-Hao Lin, Ting Ling, Si-Min Xu, Shuo-Nan Ma, Lin Zhang, Cheng-Xu Zhou, Xiao-Jun Yan, Ji-Lin Xu

**Affiliations:** ^1^Key Laboratory of Applied Marine Biotechnology, Ministry of Education of China, Ningbo University, Ningbo, China; ^2^Collaborative Innovation Center for Zhejiang Marine High-Efficiency and Healthy Aquaculture, Ningbo University, Ningbo, China

**Keywords:** *Isochrysis galbana*, *Alteromonas macleodii*, RNA-Seq, co-culture, interaction

## Abstract

Phycospheric bacteria may be the key biological factors affecting the growth of algae. However, the studies about interaction between *Isochrysis galbana* and its phycospheric bacteria are limited. Here, we show that a marine heterotrophic bacterium, *Alteromonas macleodii*, enhanced the growth of *I. galbana*, and inhibited non-photochemical quenching (NPQ) and superoxide dismutase (SOD) activities of this microalgae. Further, we explored this phenomenon *via* examining how the entire transcriptomes of *I. galbana* changed when it was co-cultured with *A. macleodii*. Notable increase was observed in transcripts related to photosynthesis, carbon fixation, oxidative phosphorylation, ribosomal proteins, biosynthetic enzymes, and transport processes of *I. galbana* in the presence of *A. macleodii*, suggesting the introduction of the bacterium might have introduced increased production and transport of carbon compounds and other types of biomolecules. Besides, the transcriptome changed largely corresponded to reduced stress conditions for *I. galbana*, as inferred from the depletion of transcripts encoding DNA repair enzymes, superoxide dismutase (SOD) and other stress-response proteins. Taken together, the presence of *A. macleodii* mainly enhanced photosynthesis and biosynthesis of *I. galbana* and protected it from stress, especially oxidative stress. Transfer of fixed organic carbon, but perhaps other types of biomolecules, between the autotroph and the heterotroph might happen in *I. galbana*-*A. macleodii* co-culture. The present work provides novel insights into the transcriptional consequences of *I. galbana* of mutualism with its heterotrophic bacterial partner, and mutually beneficial associations existing in *I. galbana*-*A. macleodii* might be explored to improve productivity and sustainability of aquaculture algal rearing systems.

## Introduction

How environmental factors affect the growth of microalgae is always a concern of researchers. Environmental factors such as illumination, salinity, temperature, and nutrient salts all have significant effects on the growth of microalgae (Suthar and Verma, 2018; Cao et al., 2020; Ananthi et al., 2021; Corredor et al., 2021). In recent years, many researchers have found that the growth of microalgae is not only affected by the abiotic environmental factors, but also the influence of biological factors such as bacteria on the growth of microalgae cannot be neglected. The complex relationship between bacteria and microalgae has attracted more and more attention (Amin et al., 2012b; Seymour et al., 2017; Lian et al., 2018; Zhang et al., 2020), becoming one of the hot spots in the field of algae research at present. Current studies have shown that bacteria can promote the growth of microalgae in various ways. For example, bacteria can release the growth-promoting hormone indole-3-acetic acid (Amin et al., 2015; Dao et al., 2018), provide essential vitamins to algae (Croft et al., 2005; Cooper et al., 2019), scavenge reactive oxygen species (ROS) (Morris et al., 2008, 2011), promote algal assimilation of iron (Amin et al., 2009, 2012a), or metabolically transform compounds released by autotrophs in ways that can impact the entire community (Durham et al., 2015; Christie-Oleza et al., 2017). Such studies have begun to investigate which factors drive algal-bacterial interactions, yet there are many gaps in our understanding of these processes. Understanding these interactions requires studying them at different scales: identifying transcriptional changes that occur when organisms interact being the most fundamental, as this is where the cell-to-cell “recognition” is first expressed.

*Isochrysis galbana* is one of the most important bait microalgae in aquaculture. It is small in body size, rich in polysaccharides, carotene and lipids with high energy, especially unsaturated fatty acids docosahexaenoic acid (DHA) and eicosapentaenoic acid (EPA), which are needed for the growth and development of shellfish. Besides, it has the characteristics of having no cell wall and being easily digested and absorbed by aquatic animal larvae (Cao et al., 2019). It is not only a good bait for aquaculture seedling, but also an important raw material for the development of bioactive substances. Meanwhile, it is also considered as one of the microalgae most likely to be industrialized. However, the cultivation of *I. galbana* is difficult in the actual production, because factors causing the bottleneck of biomass yield are still unclear. Optimal cultivation conditions of *I. galbana* should always take its associated bacterial community into account. However, studying how co-occurring bacteria affect *I. galbana* is still in its in-fancy. Therefore, it is urgent to excavate beneficial bacteria of *I. galbana* and study the mechanism of *I. galbana*-bacteria interaction, and beneficial bacterial strains may be supplemented as a new means to improve algal productivity and culture stability.

At present, some studies have reported some beneficial bacteria, which can promote the growth of microalgae, such as *Sulfitobacter* sp. (Amin et al., 2015), *Phaeobacter gallaeciensis* (Seyedsayamdost et al., 2011), *Rhizobium* sp. (Kim et al., 2014) and *Mesorhizobium* sp. (Kazamia et al., 2012; Wei et al., 2020). For *I. galbana*, there was an increase in algal biomass accumulation and growth rate in the presence of *Alteromonas* sp. and *Labrenzia* sp. (Sandhya and Vijayan, 2019). Interestingly, we found a bacterium also affiliated to the genus of *Alteromonas*, *Alteromonas macleodii*, that could promote the growth of *I. galbana*. However, the underlying interaction mechanism between *I. galbana* and *Alteromonas* remained unclear, which deserves further study.

In this study, we performed some physiological and biochemical experiments to evaluate the effects of *A. macleodii* on *I. galbana.* As a step toward further understanding the underpinnings of the effects, we examined the transcriptional responses of *I. galbana* to grow in co-culture with *A. macleodii*. This will provide novel insights into the transcriptional consequences of *I. galbana* of mutualism between *I. galbana* and its heterotrophic bacterial partner. Further, mutually beneficial associations existing between *I. galbana* and *A. macleodii* might be explored to improve productivity and sustainability of aquaculture algal rearing systems.

## Materials And Methods

### Algal Growth and Axenic Culture Generation

*Isochrysis galbana* was obtained from the Marine Biotechnology Laboratory of Ningbo University, China. NMB3 medium used in this study for culturing microalgae was composed of KNO_3_ (100 mg/L), KH_2_PO_4_ (10 mg/L), MnSO_4_⋅H_2_O (2.5 mg/L), FeSO_4_⋅7H_2_O (2.5 mg/L), EDTA-Na_2_ (10 mg/L), vitamin B_1_ (6 mg/L), and vitamin B_12_ (0.05 mg/L) (Yang et al., 2016). Microalgae were cultivated at a light intensity of 100 mmol photon m^–2^ s^–1^ under 23°C. Axenic *I. galbana* was maintained as described previously (Cao et al., 2019).

### Bacterial Growth, Isolation, and Classification

Bacteria were typically grown on 2216E medium incubated at 28°C with shaking at 200 r.p.m. Bacterial growth was measured by counting colony-forming units. Bacteria were isolated from exponential phase growing *I. galbana* cultures. 16S rRNA genes of the isolated bacteria were amplified using universal 16S rDNA primers (27F, 1492R). The temperature profile for Polymerase Chain Reaction (PCR) consisted of an initial incubation at 95°C for 5 min, followed by 32 cycles of 95°C for 30 s, 55°C for 1 min and 72°C for 2 min, and a final extension step at 72°C for 10 min. Purified PCR products were ligated into pMD19-T vector for sequencing. The 16S rRNA gene sequence was compared with reference sequences in the National Center for Biotechnology Information (NCBI) by Basic Local Alignment Search Tool (BLAST). Based on the result of 16S rRNA gene sequence alignment and phylogenetic analysis, one isolated bacterial strain shared 100% sequence identity to the validly named species *Alteromonas macleodii*.

### Co-culture Experiment

For co-culture experiments, single colony of *A. macleodii* was freshly plated before each experiment on 2216E agar plates. *A. macleodii* was inoculated into 2216E broth and incubated for 24 h in a shaking incubator (28°C, 180 rpm). Freshly prepared bacterial cells (OD_600_ = 0.4–0.6) were centrifuged (5,000 g for 5 min) and washed twice with sterile NMB3 medium. When axenic *I. galbana* was cultured to exponential phase (cell density of about 1 × 10^6^ cells/mL), *A. macleodii* was added into algal culture to achieve bacteria/algae ratios of 1:1, 10:1, and 50:1. The algal growth was determined daily by cell counting, and the bacterial growth was measured by counting colony-forming units. For the co-culture transcriptome experiments, treatments consisted of (1) *I. galbana* and *A. macleodii* co-culture (a bacteria/algae ratio of 50:1), (2) axenic *I. galbana*. All treatments were in triplicate. Sample names for *I. galbana* and *A. macleodii* co-culture group were Ig_Am 1, Ig_Am 2 and Ig_Am 3, and Ig 1, Ig 2 and Ig 3 were for axenic *I. galbana* group.

### RNA-seq Analysis

Cells were harvested at mid-exponential growth (100 h after inoculation for all treatments) by centrifuging. The supernatants were removed, and the bacteria and microalgae were immediately flash frozen in liquid nitrogen and later stored at −80°C. Total RNA was extracted using Trizol reagent (Invitrogen, CA, United States) according to manufacturer protocol. After the RNA test was qualified, mRNA was purified using the Ribo-zero kit to remove rRNA. The enriched mRNA was then broken into short fragments, separately. First-strand cDNA was synthesized using random hexamer primer and M-MuLV Reverse Transcriptase (RNase H^–^). To synthesize the second-strand cDNA, buffer solution, dNTPs, RNase H, and DNA polymerase I were added. The cDNA fragments were purified, end blunted, “A” tailed, and adaptor-ligated. PCR was used to selectively enrich those DNA fragments that had adapter molecules on both ends and to amplify the DNA in the library. Lastly, PCR products were purified (AMPure XP System) and library quality was assessed using an Agilent Bioanalyzer 2100 system. The generated libraries were sequenced on the Illumina HiSeq platform in Novogene Bioinformatics Technology Co., Ltd. (Beijing, China).

Clean reads were generated after removing the adaptor sequences, low-quality sequences (<Q20) and sequences shorter than 50 bp. High-quality clean reads from *I. galbana* and *A. macleodii* co-culture samples (Ig_Am 1, Ig_Am 2 and Ig_Am 3) were aligned against the assembled transcriptome of axenic *I. galbana*. The *I. galbana* transcriptome was assembled using the high-quality clean reads from the axenic *I. galbana* samples (Ig 1, Ig 2, and Ig 3) by the Trinity software as described for *de novo* transcriptome assembly (Grabherr et al., 2011). Seven public databases, including Clusters of Orthologous Groups (COG), Gene Ontology (GO), Kyoto Encyclopedia of Genes and Genomes (KEGG), NCBI non-redundant protein (Nr), NCBI non-redundant nucleotide (Nt), Protein family (Pfam) and Swiss-Prot, were used for unigene annotation. We then performed a differential expression analysis of each gene and identified genes responses to co-culturing with *A. macleodii*. Genes were considered to exhibit differential expression where the fold change in expression of co-culture compared with the axenic culture was ≥ 2, the *p* value was ≤ 0.05. Finally, we performed gene enrichment analysis with the corresponding database (GO enrichment:^1^; KEGG enrichment:^2^) for the differentially transcribed genes. The transcriptomic data has been deposited in NCBI Sequence Read Archive (SRA) database under the accession numbers PRJNA747627.

### Real-Time Quantitative PCR

Gene specific quantitative real-time PCR primers used in this study were documented in Supplementary Table 1. RT-qPCR was performed using SYBR Premix Ex Taq (TakaRa) on a StepOne Real-Time PCR System (ABI, United States). Relative expression levels were normalized to an actin gene and calculated using the 2^–Δ^ ^Δ^ ^*Ct*^ method as previously described (Cao et al., 2016). The significance of the differences between mean values was determined by Student’s *t*-test (*p* < 0.05).

## Results and Discussion

### *Alteromonas macleodii* Promotes the Growth of *I. galbana*

**FIGURE 1:**
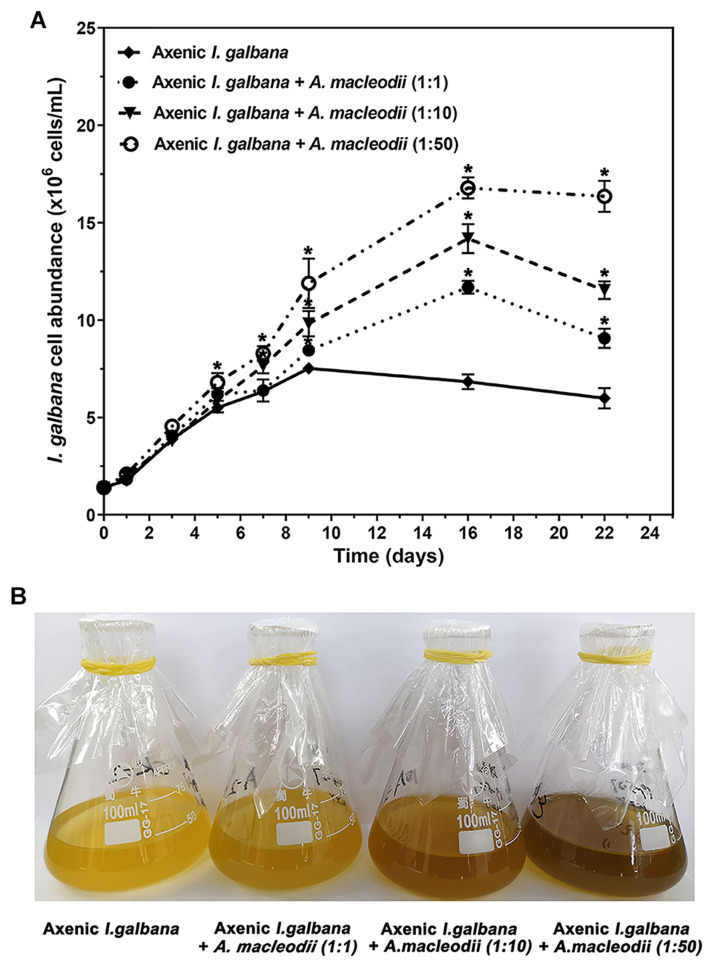
Co-cultures results of *I. galbana* and *A. macleodii* with three different initial bacteria/algae ratios of (1:1, 1:10, and 1:50) expressed as cell abundance **(A)** and the color of algal fluid **(B)**. Significance of the differences between mean values was determined with Student’s *t* test. Error bars represent standard error (SE), while asterisks (*) indicate significant difference at *p* value < 0.05.

We have analyzed the growth by cell counting for co-cultures with three different initial bacteria/algae ratio of (1:1, 1:10, and 1:50) and *I. galbana* mono-culture (Figure 1). The *I. galbana* cell abundance in co-cultures was significant higher than the control, which indicated that the growth of *I. galbana* was promoted by co-culturing with *A. macleodii*. Further, the cell abundance of *I. galbana* co-culturing with higher density of *A. macleodii* was higher (Figure 1A) and the color of algal fluid was darker (Figure 1B). We could conclude that the growth-promoting role of *A. macleodii* to *I. galbana* depends on the population density of the bacterium. As a key experimental parameter, the initial inoculation ratio has a crucial impact on the results of the co-culture system. For example, *A. macleodii* strain HOT1A3 enhanced the growth of *Prochlorococcus* at low cell densities, yet inhibited it at a higher concentration (Aharonovich and Sher, 2016).

### Physiological and Biochemical Effects of *A. macleodii* on *I. galbana*

**FIGURE 2:**
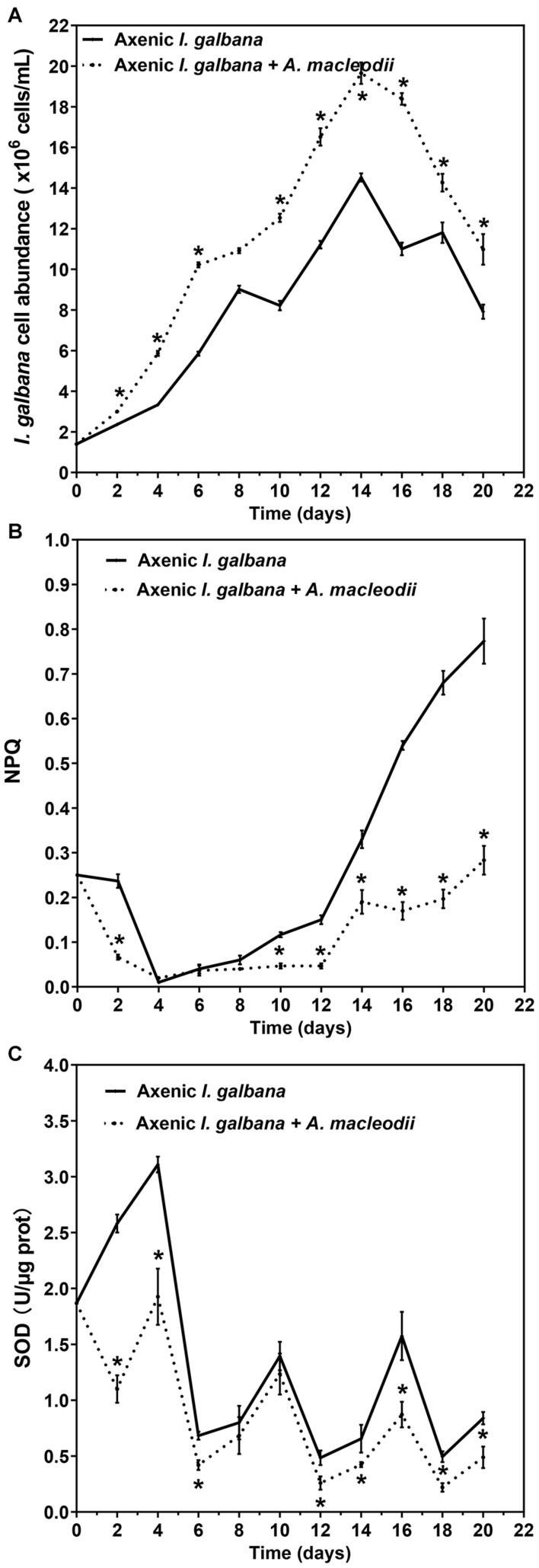
Cell abundance **(A)**, non-photochemical quenching (NPQ) **(B)** and superoxide dismutase (SOD) activity **(C)** for *I. galbana* co-culture and mono-culture during 20 d exposure time. Significance of the differences between mean values was determined with Student’s *t* test. Error bars represent standard error (SE), while asterisks (*) indicate significant difference at *p* value < 0.05.

We next focused the initial bacteria/algae ratio of 50:1, with the aim of evaluating the effects of co-culturing with *A. macleodii* on physiology and biochemistry of *I. galbana*. Same as the results shown in Figure 1, the high bacteria/algae ratio (50:1) significantly enhanced the growth of *I. galbana* (Figure 2A). Non-photochemical quenching (NPQ), a chlorophyll fluorescence parameter, reflects the ability of plant to dissipate energy, which is directly related to the ability to provide photoprotection to plant (Bachmann et al., 2004). Previous studies have documented that photosynthetic organisms would elevate NPQ when exposed to some environmental stressors (Cui et al., 2017; Zhang et al., 2017). In the present study, the values of NPQ in the co-culture group were significantly lower than that of the axenic group, especially in the later stages of co-culture (Figure 2B). Finally, co-culturing with *A. macleodii* severely inhibited the superoxide dismutase (SOD) activities of *I. galbana* comparing with the control for the entire study period (Figure 2C). It is well known that SOD plays a key role in the removal of reactive oxygen species (ROS). Interestingly, *Alteromonas* strains may scavenge ROS and thus reduce potential oxidative stress affecting *Prochlorococcus* (Morris et al., 2008, 2011). Besides, the study by Sandhya and Vijayan (2019) reported that *Alteromonas* sp. Mab 25 is able to produce extracellular antioxidants. The decreasing SOD activities in co-culture may be correlated with the scavenging of ROS by *A. macleodii*. The mechanism of *A. macleodii* promoted the growth of *I. galbana* could be related to protect it from stress, especially oxidative stress. However, additional studies are required in order to test this hypothesis.

### The Transcriptional Responses of *I. galbana* to Co-culture

To gain insight into how *I. galbana* responded physiologically to the presence of *A. macleodii*, we compared the transcriptomes of *I. galbana* in co-cultures with *A. macleodii* with in mono-culture. We next focused on the mid-exponential growth stages of *I. galbana* in co-culture, with the aim of identifying transcriptional changes in *I. galbana* that might explain the growth-promoting role of *A. macleodii* to *I. galbana* in co-culture. In general, after trimming and quality checking, clean reads% was ranged from 93.82 to 98.78 (Supplementary Table 2). The clean reads were finally combined and used to draw the transcriptome information of *I. galbana*. All data, including the error (%), Q20 (%), Q30 (%), and GC content (%), met the requirements for further analysis. Further, transcripts representing 2,157 different *I. galbana* genes were found to be differentially abundant in the co-culture, with more decreasing (1387) than increasing (770) in relative abundance (Supplementary Tables 3, 4). We next asked whether there were any pathways or molecular functions enriched in the subset of genes differentially expressed between co-cultures and axenic cultures, using KEGG analysis. The significant enrichment observed for the increased genes was “Oxidative phosphorylation,” “Photosynthesis,” “Ribosome,” “Tyrosine metabolism,” “Isoquinoline alkaloid biosynthesis” and “Carbon fixation in photosynthetic organisms,” while the depleted KEGG categories included “Steroid biosynthesis,” “Non-homologous end-joining,” “Protein processing in endoplasmic reticulum,” “Arginine biosynthesis,” “Biotin metabolism,” “alpha-Linolenic acid metabolism” and “Carotenoid biosynthesis” (Table 1).

**TABLE 1:** *Isochrysis galbana* pathways and selected KEGG categories significantly enriched or depleted in co-culture.

**Pathway**	**Genes with significantly differentially abundant transcript levels**	***P* value**
**Increased abundance during co-culture**		
Oxidative phosphorylation	*COX1, COX2, COX3, ND1, ND2, ND3, ND4, ND5, and ATP6*	1.41E-17
Photosynthesis	*psbA, psbD, psbF, psaD, psbL, psaC, and psaL*	2.88E-11
Ribosome	*RP-S2, RP-S5, RP-S10, RP-S12, RP-S15, RP-S24, RP-S27, RP-S29, and RP-L18*	2.05E-03
Tyrosine metabolism	*GOT1, s-glutathione dehydrogenase*	3.47E-02
Isoquinoline alkaloid biosynthesis	*GOT1*	3.98E-02
Carbon fixation in photosynthetic organisms	*GOT1, PGK, rpiA, rbcS, and sedoheptulose-1,7-bisphosphatase*	4.13E-02
**Decreased abundance during co-culture**		
Steroid biosynthesis	*TGL4, DHCR7, and CYP51*	1.55E-02
Non-homologous end-joining	*PRKDC, DNL4*	1.73E-02
Protein processing in endoplasmic reticulum	*SIL1, EDEM2, MAN1, HRD1, SEC23, HUGT, STT3, PDIA6, and SEC24*	1.85E-02
Arginine biosynthesis	*argE, argAB, argH, glnA, URE, and GPT*	1.96E-02
Biotin metabolism	*BIO3-BIO1, bioB*	1.97E-02
alpha-Linolenic acid metabolism	*TGL4*	2.34E-02
Carotenoid biosynthesis	*phytoene desaturase, lycopene beta cyclase, and zeaxanthin epoxidase*	3.17E-02

*COX, Cytochrome c oxidase; ND, NADH dehydrogenase; RP, Ribosomal protein; GOT1, Glutamic oxaloacetic transaminase 1; PGK, Phosphoglycerate kinase; TGL, Triacylglycerol lipase; DHCR7, 7-dehydrocholesterol reductase; CYP51, Sterol 14 alpha-demethylase; PRKDC, DNA-dependent protein kinase catalytic subunit; DNL4, DNA ligase 4; EDEM2, ER degradation enhancer; mannosidase alpha-like 2; MAN1, Mannosyl-oligosaccharide alpha-1;2-mannosidase; HRD1, HMG-CoA reductase degradation 1; PDIA6, Protein disulfide-isomerase A6; URE, Urease; GPT, Glutamic-pyruvic transaminase; BIO, Biotin.*

**FIGURE 3:**
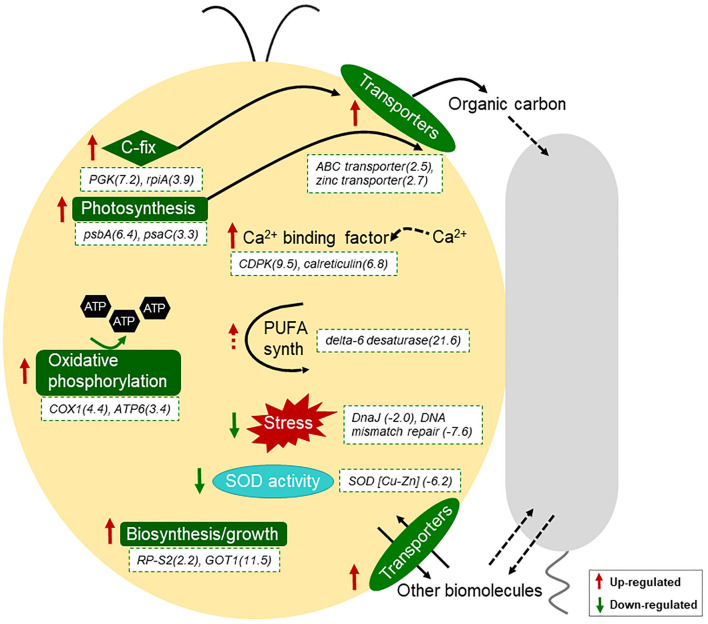
Schematic diagram of transcriptional responses of *I. galbana* to co-culture with *A. macleodii*. *I. galbana* cell in yellow and *A. macleodii* cell in gray. Red and green arrows indicate the related genes or pathways were up-regulated and down-regulated, respectively. Log_2_^*fold change*^ in comparison group Ig_Am/Ig for the expression of the related genes and the gene names were denoted with the white dotted box.

Kyoto Encyclopedia of Genes and Genomes enrichment results suggested that photosynthesis of *I. galbana* were promoted in the presence of *A. macleodii* (Table 1 and Figure 3). A similar increase in expression of these pathways was also observed in *Prochlorococcus-Alteromonas* co-cultures (Biller et al., 2016). Why might the presence of *A. macleodii* trigger a change in the photosystems of *I. galbana*? Although mechanistic insights require further research, one possible explanation is that the heterotroph, by consuming some form of dissolved organic carbon released by *I. galbana*, might have either directly or indirectly stimulated *I. galbana* to increase the production and export of carbon compounds. As organic carbon was not provided in the medium, the success of *I. galbana* and *A. macleodii* co-culture reflects the ability of *A. macleodii* to survive using the organic carbon produced by *I. galbana*. The increase in expression of carbon fixation related genes in *I. galbana* proved this to some extent (Table 1 and Figure 3). Besides, oxidative phosphorylation is one kind of way to generate energy in the form of ATP and it was activated in the presence of *A. macleodii* here (Table 1 and Figure 3). Increased energy could be used for biosynthesis, transportation and other activities. Finally, consistent with the increased growth of co-cultured *I. galbana*, we found that co-cultured cells were enriched for transcripts encoding a number of proteins involved in biosynthesis or growth (Table 1 and Figure 3). These included increased in transcripts for multiple ribosomal proteins, as well as enzymes related to Isoquinoline alkaloid biosynthesis and Tyrosine metabolism. This suggested that *I. galbana* cells were increasing their biosynthetic capability in co-culture, and the increased biosynthesis need more energy. Taken together, we hypothesized that increasing photosynthesis of *I. galbana* in co-culture will produce more energy-storing materials such as carbohydrate that could be used for respiration to produce more energy for biosynthesis, which could explain for the increased growth of co-cultured *I. galbana* in the presence of *A. macleodii*.

Besides the above enriched pathways or molecular functions, many of the transcriptional responses of *I. galbana* can be associated with transport processes under co-culture conditions (Supplementary Table 3). For example, the expression of a intraflagellar transport protein 140-like protein (Cluster-1913.45461), a ABC transporter (Cluster-1913.54562), a putative zinc transporter (Cluster-1913.58709) and sodium potassium calcium exchanger 4 (Cluster-1913.5747) was significantly increased in the presence of *A. macleodii* (Supplementary Table 3). Similar results were found in *Synechococcus*–*Shewanella* co-culture, as well as *Prochlorococcus*–*Alteromonas* (Beliaev et al., 2014; Biller et al., 2016). These results suggested that many types of biomolecules, including fixed organic carbon, might be exchanged between the co-cultured autotroph and heterotroph. The differential expression of 10 genes annotated as calcium-dependent protein kinase (CDPK), calcium-dependent protein or calreticulin (8 upregulated, 2 downregulated) suggests similarities with plant recognition of bacteria using Ca^2+^ as a second messenger (Vadassery and Oelmüller, 2009). Calcium-dependent signaling followed by cell death was described previously for nutrient-limited diatoms (Vardi et al., 2006). Our results, as well as the results reported by Durham et al., 2017 extend this by suggesting that second messenger also plays a role in relaying information on biotic stimuli. Interestingly, the expression of a delta-6 fatty acid desaturase (Cluster-1913.57529) was enrich significantly (Log_2_^*FoldChange*^ = 21.583) (Supplementary Table 3). Delta-6 fatty acid desaturase is a key enzyme for polyunsaturated fatty acids (PUFA) biosynthesis. If the presence of *A. macleodii* indeed increased markedly the polyunsaturated fatty acids content in co-culture is worth of making further research.

Finally, additional transcriptional responses of *I. galbana* were consistent with a generalized reduction in stress in the presence of the heterotroph. For example, multiple DNA repair enzymes, including DnaJ (Cluster-1913.40877), DNA mismatch repair protein (Cluster-1913.37455, Cluster-1913.28067), deoxyribodipyrimidine photolyase (Cluster-1913.105678), RNA helicase nonsense mRNA reducing factor (Cluster-1913.102129), lon protease homolog 1 (Cluster-1913.17497), REV1 Deoxycytidyl transferase (Cluster-1913.59724) were transcriptionally depleted in co-culture (Supplementary Table 4). Besides, the expression of copper/zinc superoxide dismutase (Cluster-1913.101311) was down-regulated in co-culture, which was consistent with the decreased activity of SOD (Figure 3 and Supplementary Table 4). In addition, other stress-response transcripts encoding heat shock protein 90, multiple nfx1-type zinc finger-containing proteins and some ubiquitination-related proteins also decreased in relative abundance (Supplementary Table 4). These results suggested the growth-promoting role of *A. macleodii* to *I. galbana* might be partly related to protect it from stress, especially oxidative stress, which was similar with the case in *Alteromonas*-*Prochlorococcus* interaction (Morris et al., 2008, 2011).

However, the most significant fraction of genes differentially expressed in co-culture compared with axenically growing cells have no known function. The lack of annotation hampers the interpretation of many of the responses observed and this highlights how much we have yet to learn about the mechanisms and molecules underlying *I. galbana*- bacteria interactions. Additionally, correlation analysis of the transcriptional responses of *A. macleodii* to co-culture with the responses of *I. galbana* was important to provide insight into *I. galbana* and *A. macleodii* interactions, which deserves further study. Finally, it is important to recognize that changes in relative transcript abundance within *I. galbana* and *A. macleodii* does not necessarily always lead to similar changes in protein abundance. Thus, the changes described here represent a first step toward understanding the interactions between *A. macleodii* and *I. galbana*.

### Validation of Significant Differentially-Expressed Genes by RT-qPCR

**FIGURE 4:**
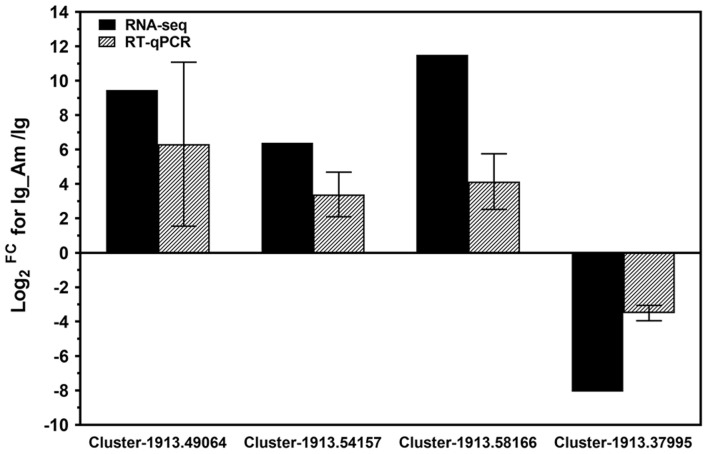
Experimental confirmation of RNA-seq data on relative fold change level of the selected differentially-expressed genes (DEGs) by real-time quantitative PCR (RT-qPCR) analyses. The results from RNA-Seq and RT-qPCR, are shown by bars with different patterns. The selected DEGs included calcium-dependent protein (Cluster-1913.49064), photosystem II protein D1 (Cluster-1913.54157), aspartate aminotransferase (Cluster-1913.58166) and biotin synthase (Cluster-1913.37995) for *I. galbana*. RT-qPCR data represent the mean ± standard error (SE) of three independent experiments.

In order to further validate the RNA-seq data, four DEGs from comparison group (Ig_Am vs Ig) were randomly selected for expression profile analyses by RT-qPCR. All these DEGs included calcium-dependent protein (Cluster-1913.49064), photosystem II protein D1 (Cluster-1913.54157), aspartate aminotransferase (Cluster-1913.58166) and biotin synthase (Cluster-1913.37995) for *I. galbana*. The results of RT-qPCR revealed that most of these DEGs shared similar expression tendency with those from RNA-seq data (Figure 4). Although there were some quantitative differences between the two analytical platforms, the similarities between these two methods suggested that the RNA-seq data were reproducible and reliable.

## Data Availability Statement

The transcriptomic data has been deposited in NCBI Sequence Read Archive (SRA) database under the accession numbers PRJNA747627.

## Author Contributions

J-LX coordinated the project. Y-YW, M-NW, Z-YK, J-HL, TL, S-MX, and J-YC conducted the experiments. J-YC designed and performed the bioinformatics and statistical analysis. S-NM, LZ, and C-XZ helped in analyzing and modeling the data. J-LX and X-JY conceived of the study and participated in its design and coordination. J-LX and J-YC prepared and revised the manuscript. All authors had read and approved the final manuscript.

## Conflict of Interest

The authors declare that the research was conducted in the absence of any commercial or financial relationships that could be construed as a potential conflict of interest.

## Publisher’s Note

All claims expressed in this article are solely those of the authors and do not necessarily represent those of their affiliated organizations, or those of the publisher, the editors and the reviewers. Any product that may be evaluated in this article, or claim that may be made by its manufacturer, is not guaranteed or endorsed by the publisher.

## Footnotes

1
http://geneontology.org/

2
http://www.genome.jp/kegg/
